# Non-anti TNF biologic modifier drugs in non-infectious refractory chronic uveitis: the current evidence from a systematic review

**DOI:** 10.1186/1546-0096-12-S1-P120

**Published:** 2014-09-17

**Authors:** Gabriele Simonini, Rolando Cimaz, Gareth T Jones, Gary J Macfarlane

**Affiliations:** 1 Rheumatology Unit-Dpt of Padiatrics University of Florence-Anna Meyer Children Hospital, Firenze, Italy; 2 Musculoskeletal Research Programme (Epidemiology Group), Institute of Applied Health Sciences, University of Aberdeen, Aberdeen, UK

## Introduction

Non-infectious chronic uveitis is a serious and disabling sight-threatening disease accounting for up to 10% of pathologies leading to blindness. Currently, a step-by step escalating immunosuppressive therapy is generally used, in children as well as in adults, and anti-TNFa biologic therapies have markedly increased the treatment options for sight-threatening uveitis refractory to conventional immune-modulatory therapy (DMARD) in addition to topical and/or systemic corticosteroids. However, a subset of patients fails to respond to TNFa blockers or is unable to tolerate these therapies and may therefore benefit from switching to another drug. In this clinical setting, the large availability of several different molecules, mostly off-label, poses the clinical question if it can be useful and safe to administer another class of biologic drugs, such as Abatacept or Rituximab, for patients with refractory auto-immune uveitis.

## Objectives

To summarize the evidence regarding the effectiveness and the safety of switching to a Non anti-TNF biologic modifier immunosuppressant treatment (NTT) currently available in clinical practice

## Methods

Acomprehensive systematic review was undertaken involving a literature search between January 2000 and April 2013 was conducted using EMBASE, Ovid MEDLINE, Evidence Based Medicine Reviews–ACP Journal Club, Cochrane libraries, and EBM Reviews. Studies investigating the efficacy of NTT as biologic modifier immunosuppressant medication for autoimmune chronic uveitis, refractory to topical and/or systemic steroid therapy, were eligible for inclusion. The primary outcome measure was the improvement of intraocular inflammation, as defined by the SUN working group criteria. We determined a combined estimate of the proportion of subjects responding to NTT.

## Results

We initially identified 526 articles, of which 89 were potentially eligible. From the selection process, a total of 10 retrospective chart reviews and 1 randomized single-blind controlled study, providing a total of 12 children and 34 adults, were deemed eligible. The studies were related to Rituximab (n=3), Abatacept (n=3), Tocilizumab (n=3) and single studies on Alemtuzumab and Anakinra. Before the NTT treatment, all the eligible subjects received several combinations of one or more DMARD and at least one anti-TNF strategy. Considering the observational studies, thus excluding 7 adults enrolled in the RCT, 8 children out 12, and 18 adults out of 27 responded to NTT treatment: 0.66 was the combined estimate of the proportion of subjects improving on NTT treatment for children (95% CI: 0.58-0.81) and adults (95% CI: 0.64-0.79). Further statistical comparison between different NTT strategies was not possible due to the small sample sizeThe only RCT reported a success rate of 2 out of 7 adult Behçet’s disease patients with a 6-month exposure to Rituximab

## Conclusion

Although randomized controlled trials are needed, the available evidence suggests that a NTT strategy may be useful in selected categories of autoimmune chronic uveitis in adults as well in childhood, refractory to a previous course of immunosuppressive treatment, both with DMARDs and anti-TNFa.

## Disclosure of interest

None declared.

